# Supplementary material for: Structures and broad-spectrum growth-inhibiting activity of formomarinobactin, formylated marinobactin analogs from the Pseudomonas lutea group
Source: Front Microbiol. 2026 Mar 13;17:1788039. doi: 10.3389/fmicb.2026.1788039 (PMC13037486; doi:10.3389/fmicb.2026.1788039)
Supplement: Supplementary file 1 [file Supplementary_file_1.docx]

Supplementary Material

**Structures and broad-spectrum growth-inhibiting activity of formomarinobactin, formylated marinobactin analogues from the *Pseudomonas lutea* group**

# Grosse C.^1a^, Hughes K.^1^, Lavender M.^1^, Cornu B.^1^, Ploegaerts G.^2^, Brandt N.^1^, Matthijs S.^1*^

^1^Unité de Recherche Produits naturels de *Pseudomonas*, Institut de Recherche LABIRIS, Brussels, Belgium. ^2^Unité de Recherche Chemie durable, Institut de Recherche LABIRIS, Brussels, Belgium

[**Supplementary Table S1**. Sequence of the primers used for RT-qPCR 2](#_Toc222767270)

[**Supplementary Table S2**. Primers used for the construction of the in-frame deletion mutants Prho^T^ΔpvdLΔtrbABC and Prho^T^ΔpvdLΔtrbABCΔTWR49273 of *P. rhodesiae* LMG 17764^T^. 2](#_Toc222767271)

[**Supplementary Table S3.** NRPS adenylation domain specificity prediction using antiSMASH. 3](#_Toc222767272)

[**Supplementary Figure S1**. Relative expression of *fmbD* and *fmbG* genes with increasing Zn (left) and Ni (right) concentrations normalized against reference gene *nadB*. ……………………………...5](#_Toc222767458)

[**Supplementary Figure S2.** Mass spectrum of each formomarinobactin peak *m/z* 874, 876, 902, 904, 930 and 932 [M+H]^+^. 6](#_Toc222767459)

[**Supplementary Figure S3.** HRMS/MS Spectra of the formomarinobactins at CID 55 eV. 7](#_Toc222767460)

[**Supplementary Figure S4.** GC-MS spectrum of fatty acid methyl esters obtained for each formomarinobactin (red) and spectrum match to NIST library or standard injection (blue). 8](#_Toc222767461)

[**Supplementary Figure S5.** Thin layer chromatography of semi-purified formomarinobactin.. 10](#_Toc222767462)

[**Supplementary Figure S6.** Growth stimulation assay of pyoverdine-negative *Pseudomonas* type strains and siderophore-negative *Pseudomonas* mutants on CAA medium supplemented with the iron chelator 2,2′-bipyridine. 11](#_Toc222767463)

[**Supplementary Figure S7.** Growth inhibition assay of *P. graminis* LMG 21661^T^ (left column) and *P. bohemica* LMG 30182^T^ (right column) against *S. capitis* I, *B. zanthoxylo* RSN02*, P. aeruginosa* LMG 1242^T^, *Enterobacteriaceae* sp. DV4951 and *E. hirae* LMG 10274 (rows). 12](#_Toc222767464)

[**Supplementary Figure S8.** Growth inhibition of formomarinobactins against *S. capitis* I, *B. zanthoxylo* RSN02*, P. aeruginosa* LMG 1242^T^, *Enterobacteriaceae* sp. DV4951, *E. hirae* LMG 10274 and *C. albicans* IHEM 3731. 13](#_Toc222767465)

Supplementary Table S1. Sequence of the primers used for RT-qPCR.

| **Gene** | **Forward primer** | **Reverse primer** |
| --- | --- | --- |
| *fmbD* | 5’-AGCGACAACGAGCTGTTCA-3’ | 5’-TCCAGCGCCTGTTCGATGT-3’ |
| *fmbG* | 5’-AGCACCAGTTCAACGACA-3’ | 5’-TGCCAAAACCGGCGTTAT-3’ |
| *nadB* | 5’-CCAGCACGACGTATTGGTA-3’ | 5’-CGTTCGCCAGATCACCTT-3’ |

**Supplementary Table S2**. Primers used for the construction of the in-frame deletion mutants Prho^T^ΔpvdLΔtrbABC and Prho^T^ΔpvdLΔtrbABCΔTWR49273 of *P. rhodesiae* LMG 17764^T^. Restriction sites are underlined.

| **Primer name** | **Primer sequence (5’→3’)** |
| --- | --- |
| **Construction ∆pvdL:** |  |
| Amplification of fragment A and B | |
| Prho^T^-pvdL-AF | GTGAAGCTTCATGATGGACGCCTTCGAACT |
| Prho^T^-pvdL-AR | GTGTCTAGAGAAGCGCTCCAAGGTGTCAA |
| Prho^T^-pvdL-BF | GTGTCTAGAGCCTCGACCCGTGGCACTT |
| Prho^T^-pvdL-BR | GTGGAATTCCCCTTCCAACTCCGCCATCA |
| Confirmation *pvdL* deletion mutant | |
| Prho^T^-pvdL-conF | CGACGGTCACCACTTCTTCA |
| Prho^T^-pvdL-conR | GCCATCGAGGCGTGGTATC |
| **Construction ∆trbABC:** |  |
| Amplification of fragment A and B | |
| Prho^T^-trbABC-AF | GTGAAGCTTGGCAGCTGATCGATCGATAC |
| Prho^T^-trbABC-AR | GTGTCTAGAAGGGACGATTCAGGCGTCAT |
| Prho^T^-trbABC-BF | GTGTCTAGACTTGAGTTACGCCGTGGATTG |
| Prho^T^-trbABC-BR | GTGGGATCCCCGATTTATCGAACAGGCATTG |
| Confirmation *trbABC* deletion mutant | |
| Prho^T^-trbABC-conF | GGCTCCATCGACGCCATCA |
| Prho^T^-trbABC-conR | GTGCGCGAAGAGTTGGATGA |
| **Construction ∆TWR49273:** |  |
| Amplification of fragment A and B | |
| Prho^T^-TWR49273-AF | GTGAAGCTTGGGCTATCTCGGTTGGAATC |
| Prho^T^-TWR49273-AR | GTGTCTAGAGGATGGGGAGGCGCATTG |
| Prho^T^-TWR49273-BF | GTGTCTAGATGCTCGGTACGGTGTCTTAC |
| Prho^T^-TWR49273-BR | GTGGAATTCCGACGAGTTATCCACAGTTCT |
| Confirmation TWR49273 deletion mutant | |
| Prho^T^-TWR49273-conF | TGCCTGGCAGCAATGGTTGA |
| Prho^T^-TWR49273-conR | GTGCATAACCTTACGGAACAGTT |

Supplementary Table S3. NRPS adenylation domain specificity prediction using antiSMASH. X = unassigned residue

| **Module** | **PKS/NRPS** | **Nearest Stachelhaus code** | **8Å match** | **Stachelhaus code match** |
| --- | --- | --- | --- | --- |
| MrbD | | | | |
| 1 | D-Asp | DLTKIGHVGK | 88% | 100% (strong) |
| 2 | Dab | DIWELTADDK | 88% | 100% (strong) |
| MrbE | | | | |
| 1 | D-Ser | DVWHVSLIDK | 97% | 100% (strong) |
| 2 | X = Fo-OH-Orn  X = OH-Orn | DGEVCGGVTK  DGEACGGVTK | 74%  71% | 80% (moderate) |
| 3 | Ser | DVWHVSLIDK | 97% | 100% (strong) |
| 4 | X = Fo-OH-Orn  X = OH-Orn | DGEVCGGVTK  DGEACGGVTK | 74%  71% | 80% (moderate) |

*fmbD*

*fmbG*

*fmbD*

*fmbG*

Supplementary Figure S1. Relative expression of *fmbD* and *fmbG* genes with increasing Zn (left) and Ni (right) concentrations normalized against reference gene *nadB*. Experiments were performed in triplicate. Values correspond to mean ± SEM. Brown-Forsythe and Welch ANOVA followed by Dunnett’s T3 test for multiple comparisons indicates non-significant differences between control (0 µM) and higher concentrations.

**
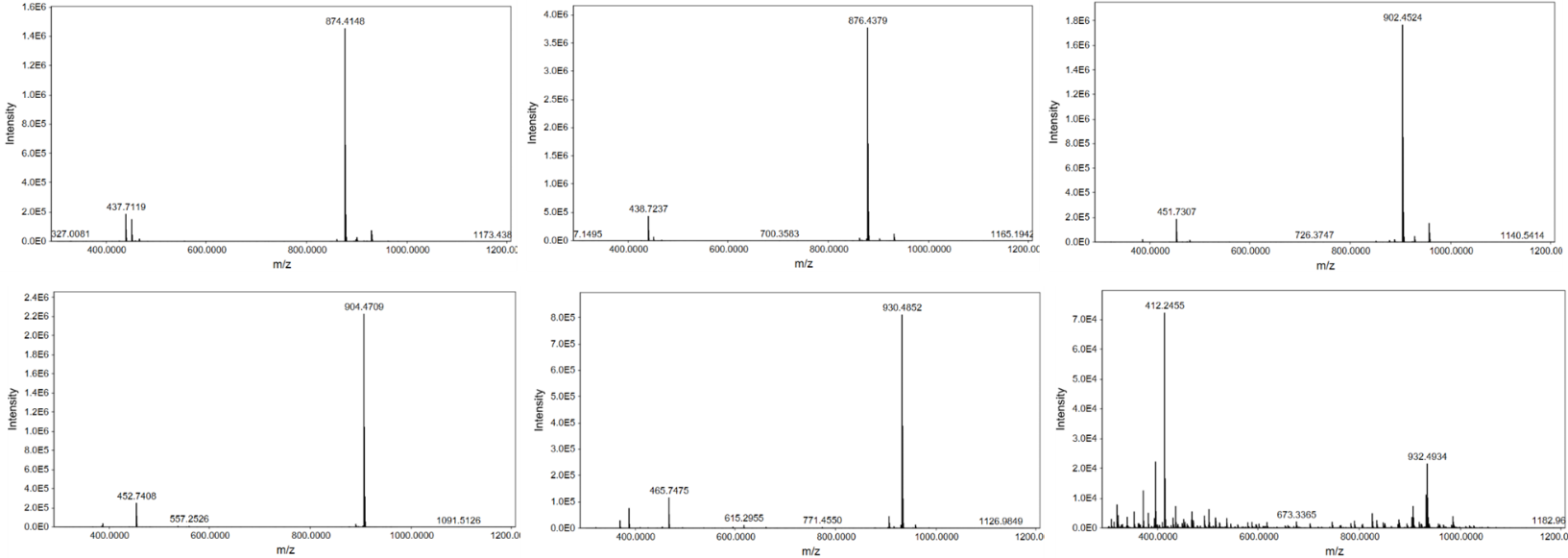
**

Supplementary Figure S2. Mass spectrum of each formomarinobactin peak *m/z* 874, 876, 902, 904, 930 and 932 [M+H]^+^.


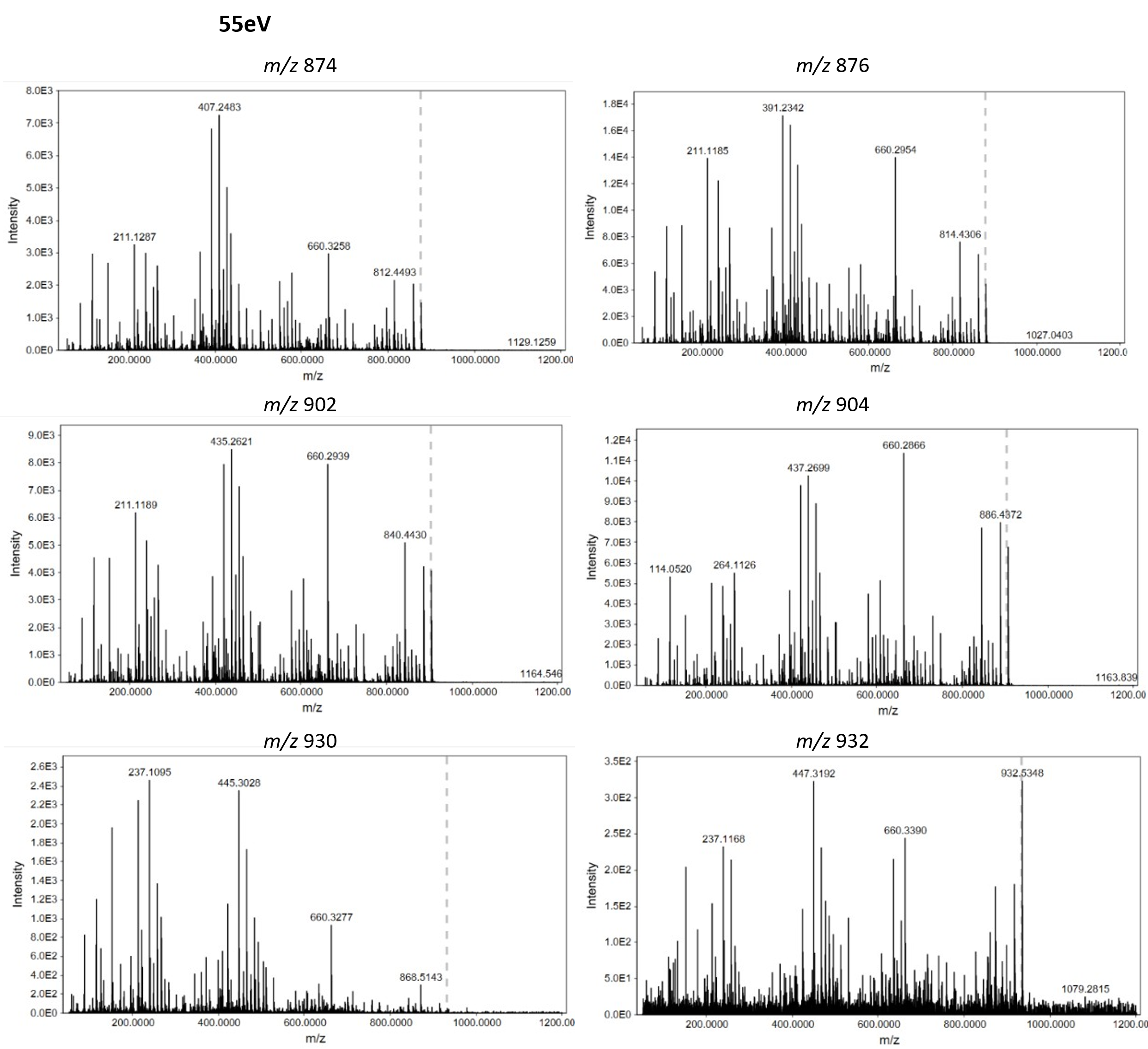


Supplementary Figure S3. HRMS/MS Spectra of the formomarinobactins at CID 55 eV. Similar fragmentation patterns are observed with a common fragment of 660 Da.


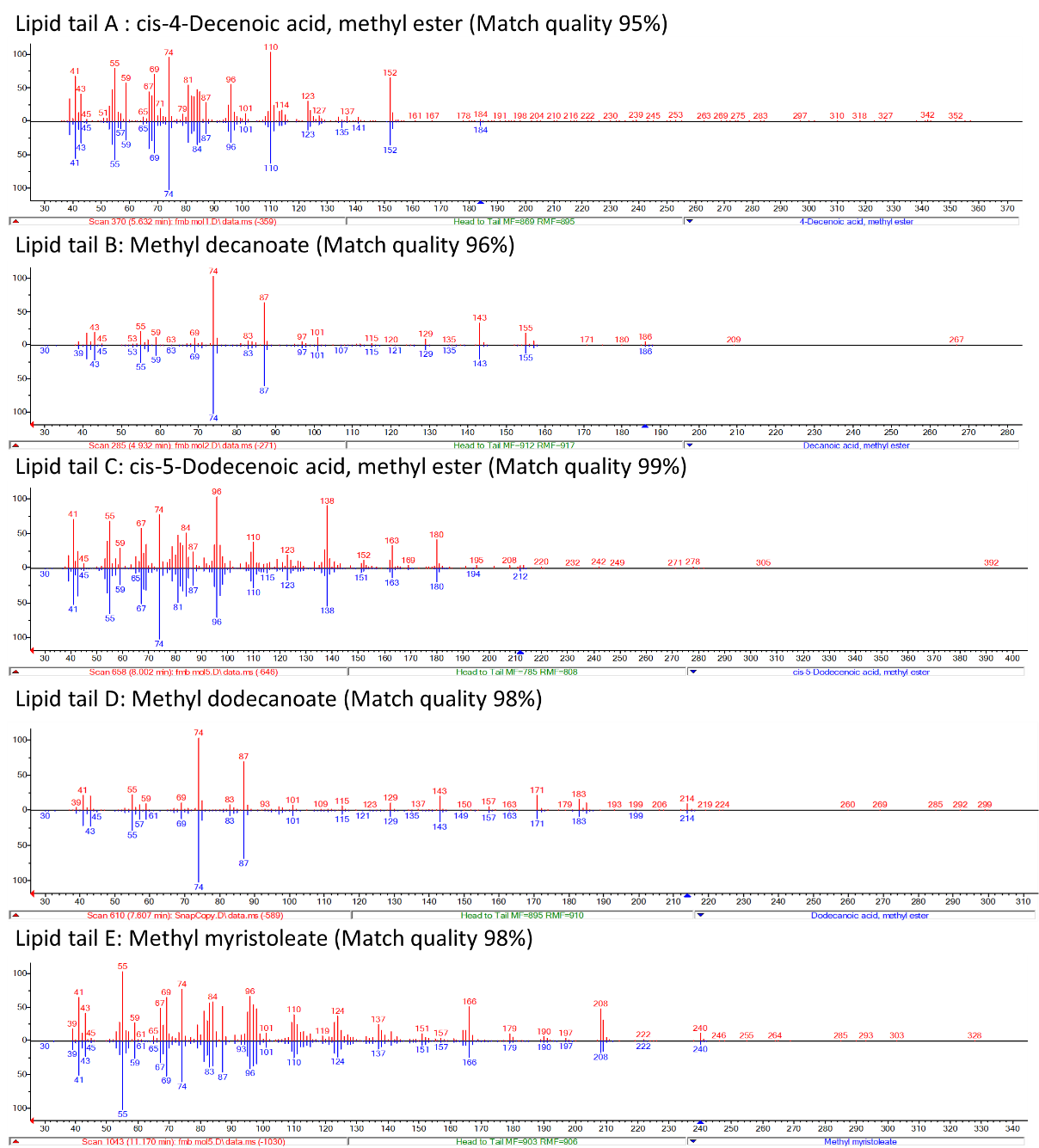
 Supplementary Figure S4. GC-MS spectrum of fatty acid methyl esters obtained for each formomarinobactin (red) and spectrum match to NIST library or standard injection (blue).


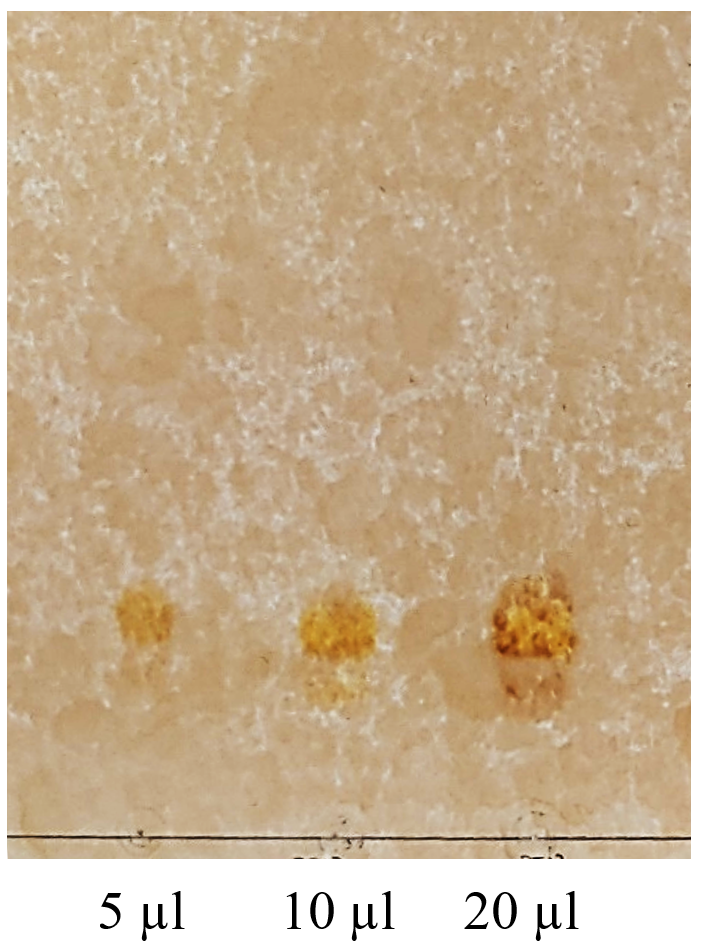


Supplementary Figure S5. Thin layer chromatography of semi-purified formomarinobactin. 5, 10 and 20 µl samples were spotted on precoated TLC silica gel 60 F254 plates (Merck) and were allowed to dry. The plates were run in an n-butanol:acetic acid:dH_2_O (12:3:5) solvent system.

Supplementary Figure S6. Growth stimulation assay of pyoverdine-negative *Pseudomonas* type strains and siderophore-negative *Pseudomonas* mutants on CAA medium supplemented with the iron chelator 2,2′-bipyridine. Pyoverdine-negative type strains: (A) *P. kuykendallii* LMG 26364ᵀ (200 µM). Siderophore-negative mutants: (B) *P. simiae* WCS417-M634 (500 µM), (C) *P. fluorescens* SBW25-15F3 (400 µM), (D) *Pseudomonas* sp. WCS374-BT1 (600 µM) and (E) *P. capeferrum* WCS358-JM213 (500 µM).


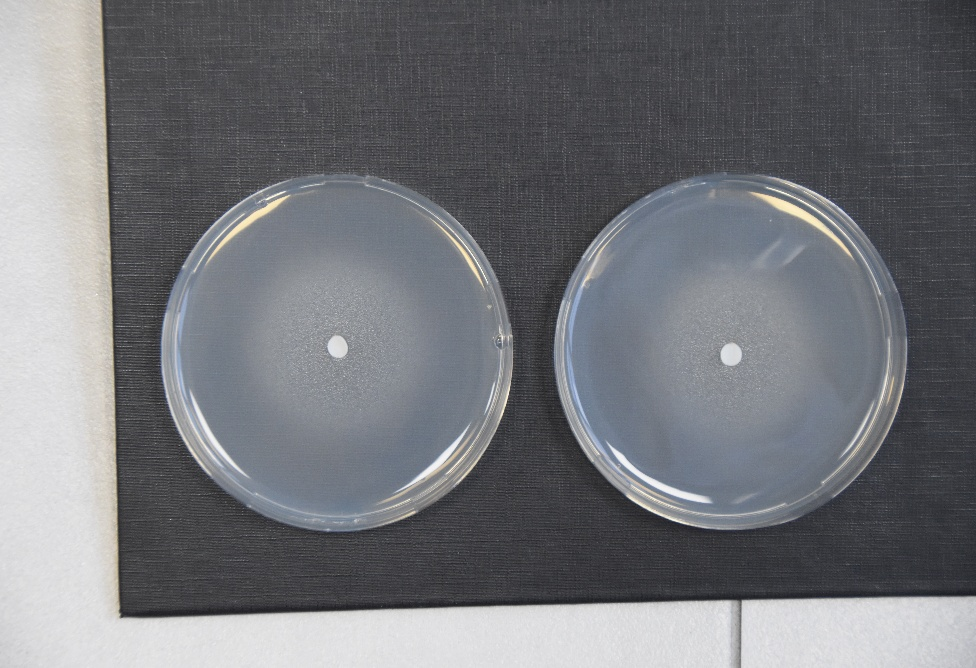


B


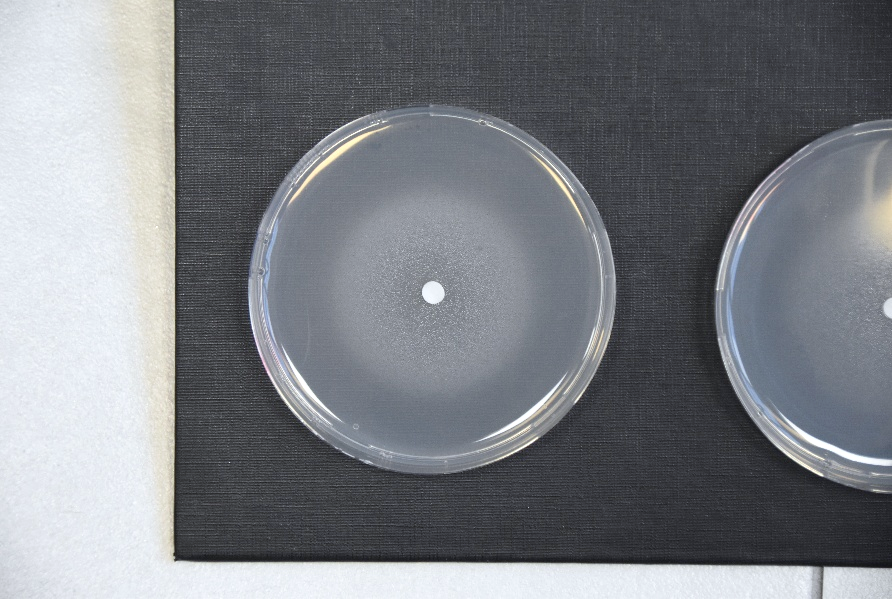


C


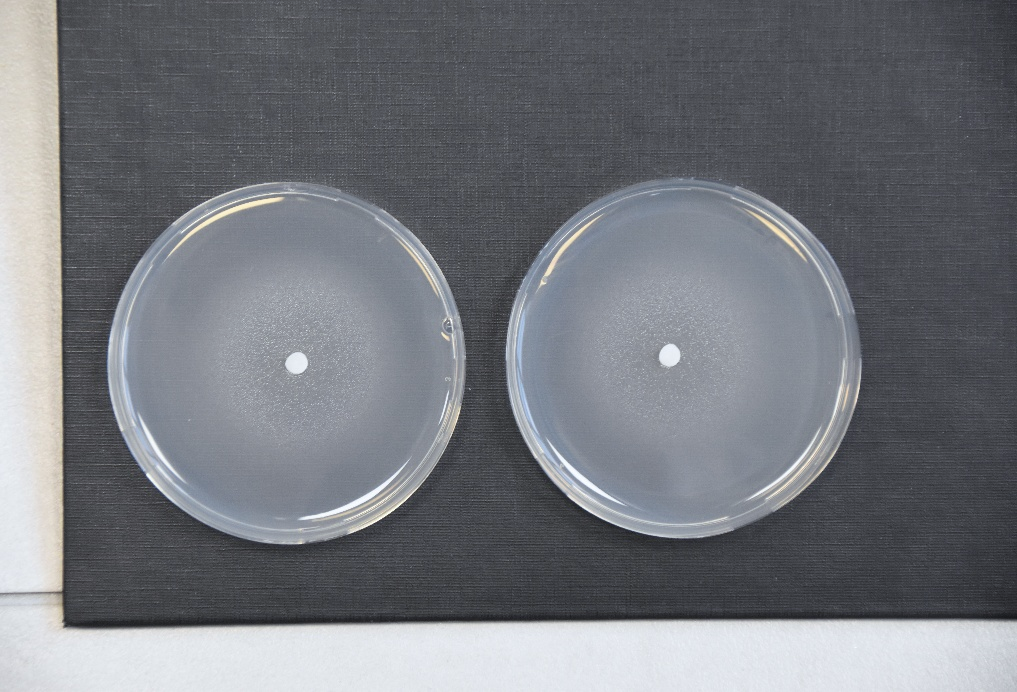


D


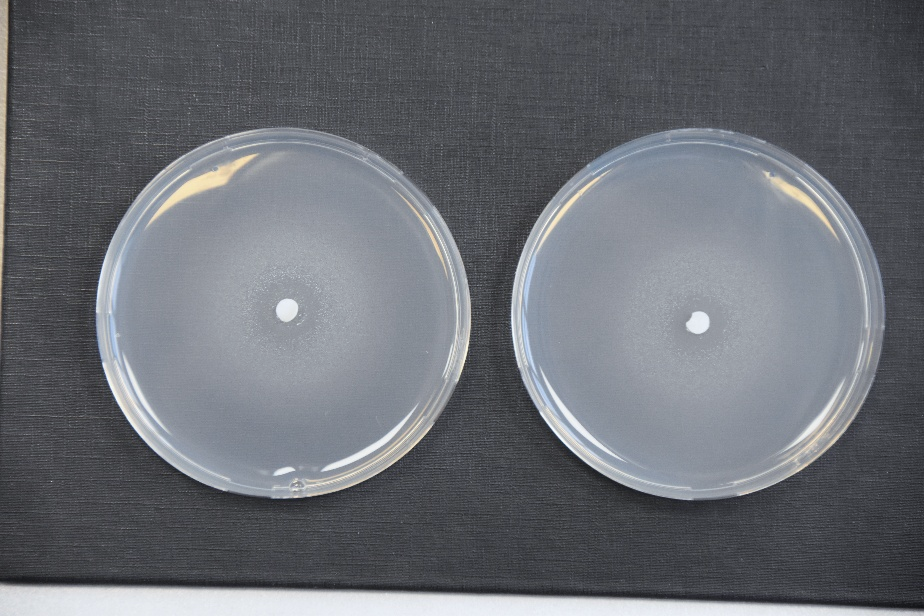


E


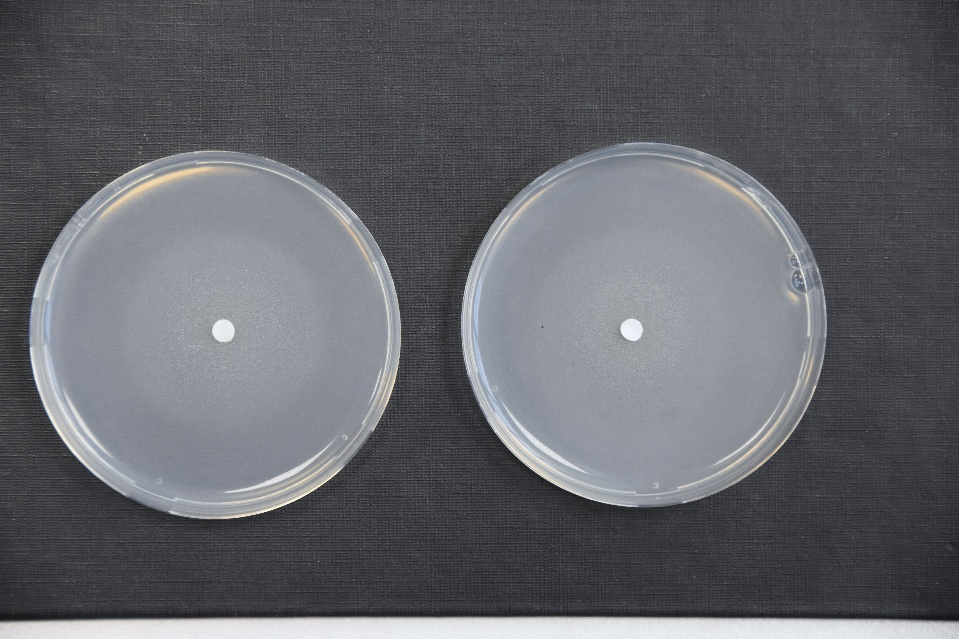


A


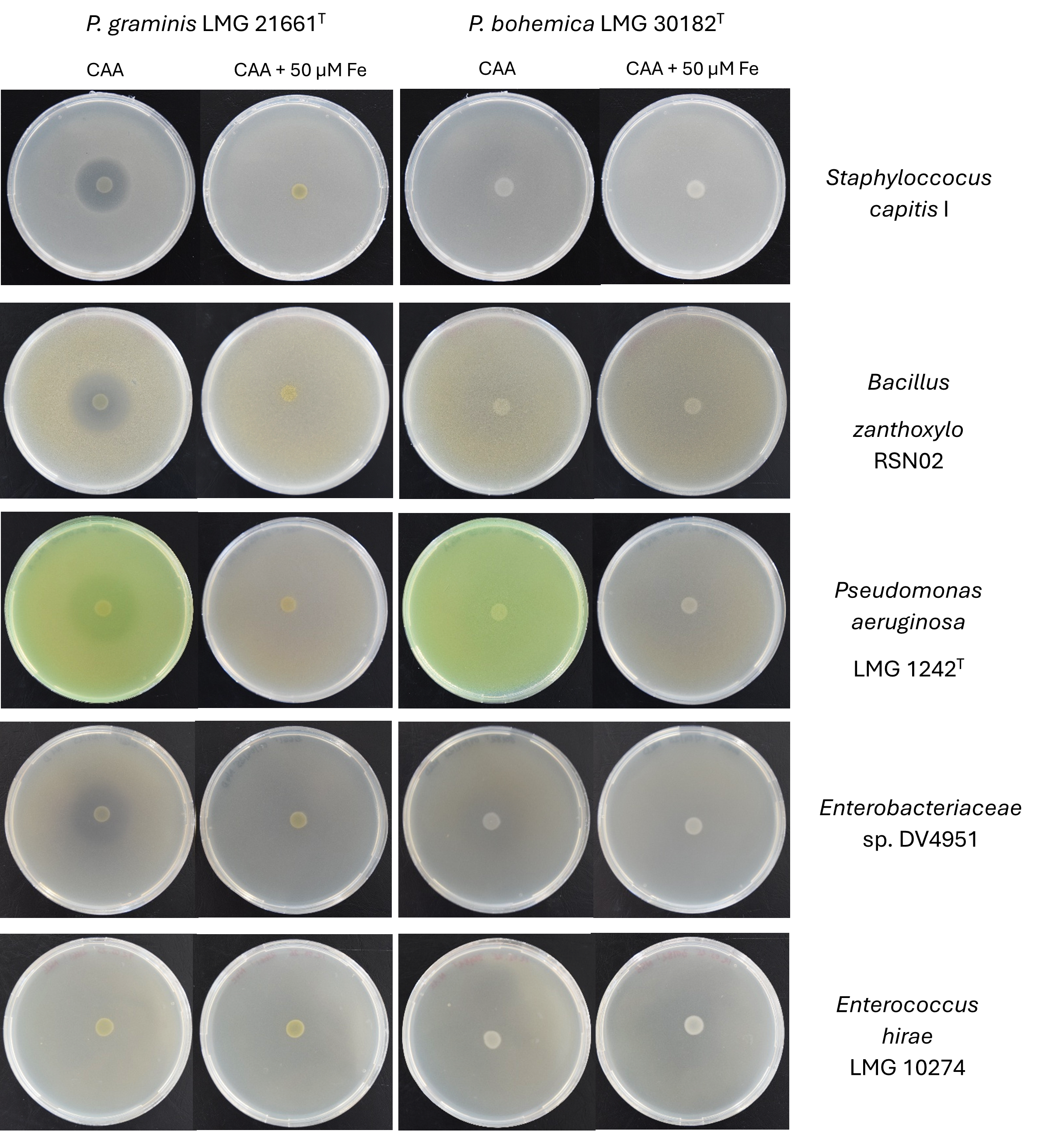


Supplementary Figure S7. Growth inhibition assay of *P. graminis* LMG 21661^T^ (left column) and *P. bohemica* LMG 30182^T^ (right column) against *S. capitis* I, *B. zanthoxylo* RSN02*, P. aeruginosa* LMG 1242^T^, *Enterobacteriaceae* sp. DV4951 and *E. hirae* LMG 10274 (rows). All images are linearly adjusted for contrast (+20%) for presentation.

**
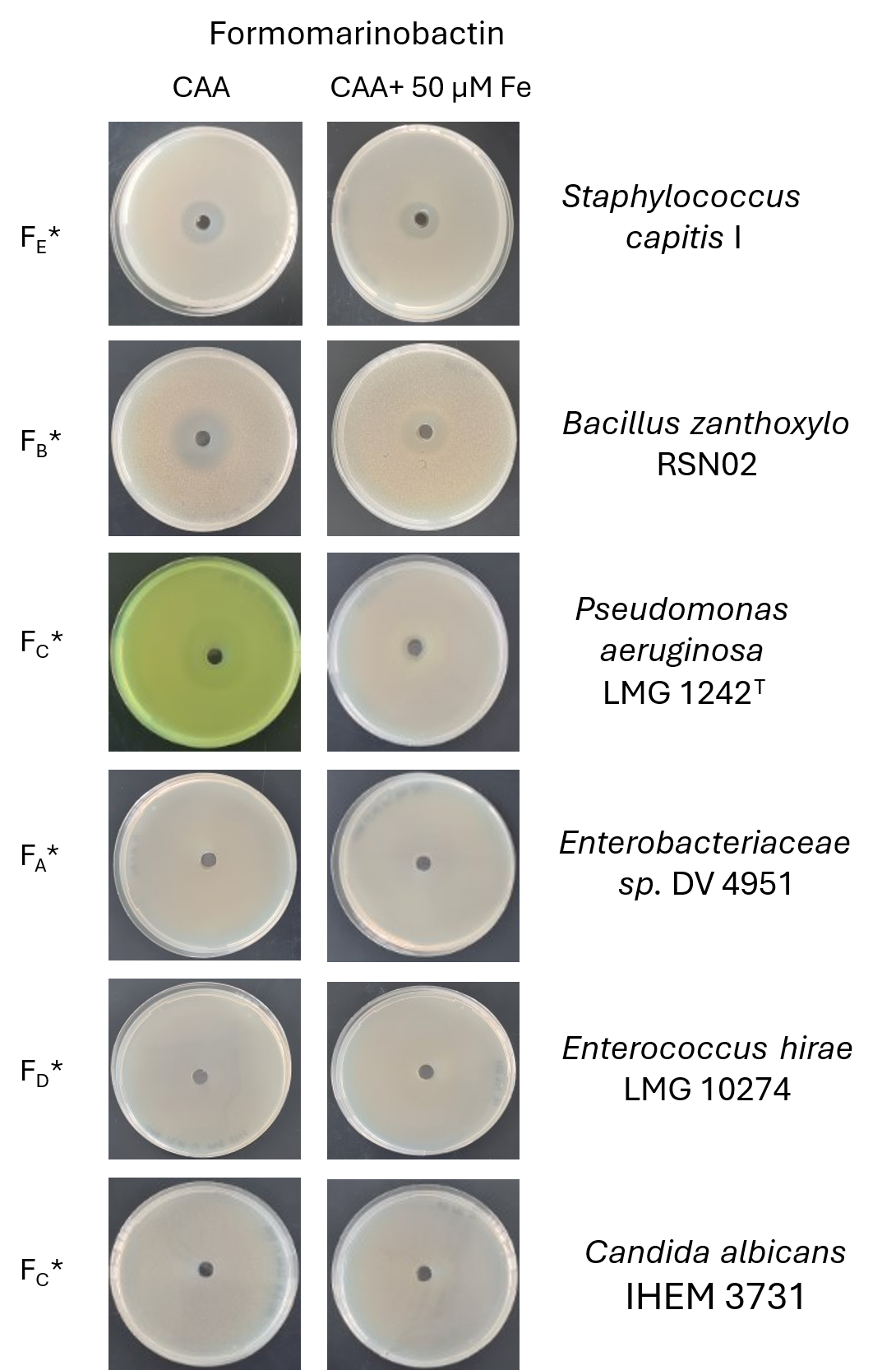
**

Supplementary Figure S8. Growth inhibition of formomarinobactins against *S. capitis* I, *B. zanthoxylo* RSN02*, P. aeruginosa* LMG 1242^T^, *Enterobacteriaceae* sp. DV4951, *E. hirae* LMG 10274 and *C. albicans* IHEM 3731. *Molecule chosen for representative picture (F_A_ = *m/z* 874, F_B_ = *m/z* 876, F_C_ = *m/z* 902, F_D_ = *m/z* 904 and F_E_ = *m/z* 930).
